# Supplementary figures and images for: Improving patient-centred counselling skills among lay healthcare workers in South Africa using the Thusa-Thuso motivational interviewing training and support program
Source: PLOS Glob Public Health. 2024 Apr 24;4(4):e0002611. doi: 10.1371/journal.pgph.0002611 (PMC11042703; doi:10.1371/journal.pgph.0002611)

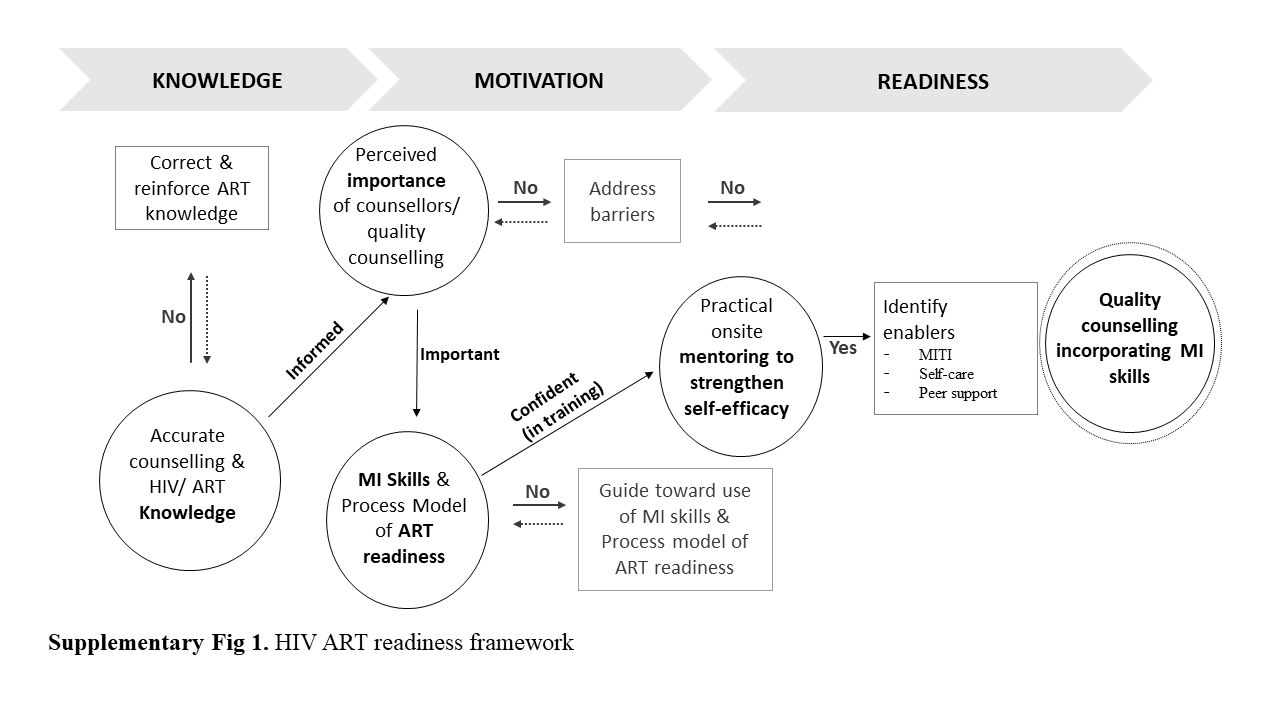

Supplement: S1 Fig — (TIF) [file pgph.0002611.s001.tif]
