# Supplementary material for: Improving patient-centred counselling skills among lay healthcare workers in South Africa using the Thusa-Thuso motivational interviewing training and support program
Source: PLOS Glob Public Health. 2024 Apr 24;4(4):e0002611. doi: 10.1371/journal.pgph.0002611 (PMC11042703; doi:10.1371/journal.pgph.0002611)
Supplement: Attachment — Submitted filename: Response to reviewers_revised submission_2.docx [file pgph.0002611.s002.docx]

**Response to reviewer comments:**

1. Please ensure that Funding Information and Financial Disclosure Statement are matched.

- *Thank you for making us aware of this oversight. We have updated the financial disclosure statement to match with the funding information.*

2. In the Funding Information you indicated that no funding was received. Please revise the Financial Discloure field to reflect funding received.

- *We have updated the funding information as indicated above.*

3. Tables should not be uploaded as individual files. Please remove these files and include the Tables in your manuscript file as editable, cell-based objects. For more information about how to format tables, see our guidelines: https://journals.plos.org/globalpublichealth/s/tables

- *We have included the tables in the manuscript file in the updated manuscript*

4. Baseline counsellor survey & MITI analysis coding.do, Baseline counsellor survey & MITI analysis log file.txt, Baseline counsellor survey data.dta & MITI analysis long.dta are currently uploaded as file type “Other”, which is not viewable by the reviewers. Please change the file type(s) to 'Supporting Information' and include a legend in the manuscript if you wish it/them to be included in review.

- *As indicated below, we would like to keep these files under the “Other” category as we have placed them in the public repository Figshare (updated link shared below) for publication.*

6. The DOI link (10.6084/m9.figshare.24891573) for the figshare repository that you shared appears to be either private or invalid. We kindly request you to provide the correct link or make your dataset publicly available. Additionally, we have noticed that you have included multiple data sharing files in the ‘Other’ category: ‘Baseline counsellor survey & MITI analysis coding.do’, ‘Baseline counsellor survey & MITI analysis log file.txt’, ‘Baseline counsellor survey data.dta’, and ‘MITI analysis long.dta’. Please be aware that files provided by the authors in the ‘Other’ category will not be published online. If you wish for these files to be published alongside your article, please move them to the ‘Supporting Information’ category. Alternatively, we can make this change on your behalf.

If these files are already included in the figshare repository and have been provided here for reference purpose only, you can provide a valid DOI link to the figshare repository and continue to keep these files in the ‘Other’ category.

- *Thank you for this feedback, this was an oversight on our part, the correct link is :* [*https://figshare.com/s/5344ffeab856882fd9fb*](https://figshare.com/s/5344ffeab856882fd9fb)*. Additionally, the files mentioned have been included in the DOI link to the Figshare repository. We would prefer that the files be kept under the "Other" category for the submission.*
